# When a bad day at the golf course is a bad day at the office: occupational stressors, institutional supports, and the mental health of NCAA golf coaches

**DOI:** 10.3389/fspor.2023.1286965

**Published:** 2023-11-30

**Authors:** Laura Upenieks, Brendan M. Ryan, Howie J. Carson

**Affiliations:** ^1^ Baylor University, Waco, TX, United States; ^2^ Independent Researcher, Tampa, FL, United States; ^3^ Moray House School of Education and Sport, The University of Edinburgh, Edinburgh, United Kingdom

**Keywords:** anxiety, burnout, depression, mental well-being, work stress

## Abstract

This study examined the mental health of NCAA collegiate golf coaches. Utilizing the person-environment fit theory and previous literature on coaches' well-being, this study examined four outcomes among 48 participants, namely: depressive and anxiety symptoms, burnout, and job turnover intentions. Results suggested that coaching stressors (e.g., administrative tasks, practice plans, pressure to win) only associate with greater burnout. More consistent evidence showed that workplace stress (e.g., lack of control and autonomy, poor work-family balance) associated with higher levels of all outcomes. Finally, greater perceived organizational support had a beneficial association with each outcome. The findings of the current study suggest golf coaches are at risk of mental health problems because of the stressors of this job. Taken as a whole, athletic departments, coaches, and student-athletes must reconsider norms that overemphasize performance and underemphasize self-care and work-life balance.

## Introduction

Given the extensive potential for cognitive and physical demands placed on collegiate coaches, it is unsurprising that this coaching context has been reported to challenge mental health and career sustainability (1, 2). Coaches are under constant pressure related to performance expectations alongside the perennial threats of possible funding cuts and job losses. Collegiate coaches often work long, irregular hours and travel extensively for recruiting and competition purposes (3). Coaches are also under institutional control, needing to be constantly available, which can lead to work-home interference (4). Scholars have noted that communicating with athletes, recruiting, travel time, administrative demands, and a lack of control are some of the stressors reported by NCAA coaches in the United States (1). Within elite sports, the typical role and expectation for the coach is to ensure athletes' performance, physical health, and well-being (5, 6) in a climate where there is tremendous pressure to win, which can inevitably place coaches' mental health at risk. Study of coach well-being and development has, therefore, become a growing area of interest (e.g., 7).

Regarding such stressors, NCAA golf coaches are an important group to study because the profession offers much lower compensation than other sports at the top tier (e.g., basketball, football). On average, a Division 1 golf coach earns $78,172 according to the U.S Bureau of Labor Statistics (8). In addition, golf coaches spend about 8–14 weeks a year away for tournaments (≈24 competition days), and many take multiple international trips a year for recruiting purposes. Golf practices can also be longer than other sports, especially if qualifying rounds to earn spots on the travel roster are being played (4–5 h or more). Golf coaches frequently correspond with specialist coaches/practitioners with whom their players work, address administrative details and fundraising issues, work with players' parents, and support typical student-athlete issues. Research also suggests that coaches in non-revenue generating sports, such as golf, were more likely to experience emotional exhaustion and increased burnout compared to coaches of revenue-generating sports of basketball and football (9). Finally, collegiate golf coaches have less autonomy than other elite level coaches (e.g., Sean Foley, Butch Harmon). Elite level coaches in golf can choose their own clients to work with and generally set their own hours. There is also a wide range of what constitutes “elite” golf (e.g., coaching talented juniors, touring professionals, etc.). The nature of the stark differences in what the day-to-day life of an elite golf coach looks like would make asking typical work-based questions difficult to tailor. Therefore, given the relative dearth of attention paid to collegiate golf coaches, this initial study seeks to explore the effects of workplace stressors and supports on the mental health of golf coaches, which as a group have never been empirically studied.

### Variegated outcomes of well-being in the golf coaching profession

This study attempts to broadly explore the well-being outcomes of coaches, which we motivate below.

#### Depressive and anxiety symptoms

It is widely accepted that sports coaching is a demanding profession (1, 10, 11), involving stressors that include working long hours (e.g., 12), insecure employment based on athletic performance (13), conflict between work and family life (14, 15), and high emotional investment (16). Moreover, coaches are required to maintain their own psychological and physical health and performance while supporting the athletes with whom they work (17). Indeed, scholars have long-recognized coaches as performers in their own right (see 18), because failure to cope effectively with demands can lead to detrimental implications for their performance and how they function in their daily lives. Moreover, while coaches have frequent contact with their athletes, many coaches experience isolation in their role and lack perceived social support (19).

Coaches also experience stressors related to the pressures of competition and the ongoing demands to win. In this environment, they feel like they always have to produce, and if they are successful, there is pressure to maintain their success (20). Other challenges related to the all-encompassing pressure to win are financial and/or concern work-life balance. Not all golf coaches, for instance, are employed on a full-time basis. Financial support and job contracts are pertinent stressors for part-time coaches. Job security, however, is often experienced as a stressor even by head coaches at the highest level (1). In addition, being judged only on the impact of expectations and evaluations from others was harmful when coaches felt they had no control over this process (21). Taken together, the combination of these stressors tends to have a detrimental impact on coaches' well-being (22). Altogether, coaching has been identified as a precarious profession because of its short-term possibility contingent on success and intense demands (see 23, 24).

#### Burnout and job turnover intentions

Maslach and Leiter (25, p. 17) describe burnout as the “index of the dislocation between what people are and what they have to do. It represents an erosion in values, dignity, spirit, and will—an erosion of the human soul.” Burnout is not classified as a mental disorder or medical condition, but burnout has been a highly researched, complex topic of study. Curiously, as noted by Freudenberger (26), committed and dedicated workers are more prone to experiencing burnout. Broadly, burnout is maladaptive and a key concern for organizations due to its impact on employee morale, well-being, performance, and turnover intentions (27).

Research on coach burnout has increased due to the rise in demands and expectations from athletic departments that have led some coaches to voluntarily leave the profession (28). Raedeke et al. (29, p. 181) offered a more precise definition of sport burnout, which is a “withdrawal from sport noted by a reduced sense of accomplishment, devaluation/resentment of sport, and physical/psychological exhaustion.” Studying burnout among coaches has become crucial because of the possible repercussions for coaches' mental health, and the possibility that the coach could negatively affect the athletes they support (30).

Within the literature on coach burnout, organizational norms and expectations have also been identified as important. There is some evidence that coach burnout may increase if a coach does not feel that their work is valued and acknowledged or does not feel that their athletic administration will support them in disputes with players or parents (31). According to Ryff and Keyes (32, p. 720), athletic administrators can promote organizational and social support to coaches to help them achieve “environmental mastery”, summarized as “the competence to manage the environment effectively to control and use it and its opportunities or to form surroundings that help one to fulfill personal needs and values.” Organizational justice is another critical component to consider, including perceptions of fairness in procedures (33). Altogether, higher levels of perceived organizational support were linked to greater job commitment, sense of belonging, and pride in an employees' organization (34). A poorer workplace climate and culture can make coaches consider leaving their job, lending itself to higher turnover intentions of coaches (35).

## Methods

### Participants

A convenience sample of 52 NCAA US golf coaches were contacted via e-mail to complete a 10-min survey (Qualtrics, USA), and 48 coaches (*M*_age_ = 45.81 ± 9.27 years; 75% male, 25% female) provided total responses for analysis (92.31% response rate). Inclusion criteria were that participants were an NCAA head coach, associate coach, or assistant coach of a golf program. While a small sample by conventional standards, our sample represents approximately 15% of collegiate golf coaches in the US and is the first study of its kind. Taken together, the sample represents each of the Power 5 NCAA conferences and involve a mix of both head and assistant golf coaches, and men and women. Almost 85% of the sample were full-time coaches, and nearly two-thirds (65.22%) coached at the NCAA Division 1 Level. Over 80% of our sample were head coaches. Prior to data collection, ethical approval was obtained from the Baylor University Institutional Review Board and all participants provided informed consent.

### Dependent variables

#### Depressive symptoms

Depressive symptoms are measured by an eight-item scale derived from the Center for Epidemiological Studies (CES-D; 36). Scores were averaged across the 8 items to form a continuous variable of depressive symptoms, where higher scores indicate greater depressive symptoms (alpha = 0.87).

#### Anxiety symptoms

Anxiety symptoms were assessed with the seven-item Hospital Anxiety and Depression Scale (HAD-S; 37). Scores were averaged across the seven items to form a continuous measure of anxiety symptoms, where greater scores indicate greater depressive symptoms (alpha = 0.83).

#### Burnout

Since collegiate coaches are highly entrenched in academic institutions and there is not a developed psychometrically sound instrument to measure coach burnout, we used the Maslach Burnout Inventory (MBI) Educators Survey. The MBI Educators Survey has three core aspects: emotional exhaustion, depersonalization, and a lack of personal accomplishment (38). The MBI Educators Survey was assessed on a 0–6 scale (0 = never, 6 = every day), with the stem of “how often…”. To ensure the survey was of reasonable length, we used the *emotional exhaustion* subscale of the MBI Educators Survey, which consists of the following eight items: (1) “I feel emotionally drained from my work,” (2) “I feel used up at the end of the workday,” (3) “I feel fatigued when I get up in the morning and have to face another day on the job,” (4) “Working with people all day is really a strain for me,” (5) “I feel burned out from my work,” (6) “I worry that this job is hardening me emotionally,” (7) “I feel frustrated by my job” and (8) “I feel I’m working too hard on my job.” Scores on these eight items were averaged to form a continuous measure of burnout, with higher scores representing greater burnout (alpha = 0.94).

#### Job turnover intention scale

The Turnover Intention Scale (TIS; 39) was developed to gauge the extent to which an employee intends to stay in their organization. We employed the TIS to assess whether occupational stressors and organizational support were associated with turnover intentions of collegiate golf coaches. The TIS-6 is a six-item scale to assess how often a person has considered, for instance, “leaving their job,” “dreaming about getting another job,” and “accepting another job at the same compensation level should it be offered to you?” in the past 9 months. A Likert scale ranging from 1 = “Never” to 5 = “Always” was used to score all responses, which were averaged across the six items. Higher scores on the TIS-6 indicated higher levels of an intention to leave an organization (alpha = 0.90).

### Focal independent variables

#### Coaching stressors scale

The measures developed by Mignano (21) were used to assess the level of coaching-specific, work-related stressors. The Coaching Stressors Scale (CSS) is a 12-item measure with the question of “How often has each of the following aspects of the coaching profession produced occupation stress within the past 12 months?” The factors included administrative tasks, fundraising, interpersonal team issues, parental factors, practice plans/competitive preparation, pressure to win, recruiting student-athlete relationships, supervisor/athletic department issues, time demands, travel, and work/personal life balance. Participants responded using a Likert scale from 1 to 7 (1 = “never,” 4 = “sometimes,” 7 = “always”). Items were averaged to form one scale of coaching stressors (alpha = 0.82).

#### Workplace stress scale

Six items gauged the culture and conditions of coaches' workplace environments. The six items were: (1) “Conditions at work are unpleasant or sometimes even unsafe,” (2) “I feel that my job is negatively affecting my physical or emotional well-being,” (3) “I have too much work to do and/or too many unreasonable demands,” (4) “I feel that my job pressures interfere with my family or personal life,” (5) “I have adequate control over my work duties” (reverse-coded), and (6) “I receive appropriate recognition or rewards for good performance” (reverse-coded). On each item, responses were scored where 1 = “never” and 5 = “very often.” Items were averaged to form a scale of workplace stress (alpha = 0.86).

#### Perceived organizational support

Participants were asked to respond to seven items that assess the support they receive from their athletic department (see 21). These items were: (1) “My athletic department values my contribution to its well-being,” (2) “My athletic department fails to appreciate any extra effort from me,” (3) “My athletic department would ignore any complaint from me,” (4) “My athletic department really cares about my well-being” (reverse-coded), (5) “Even if I did the best job possible, my athletic department would fail to notice,” (6) “My athletic department shows very little concern for me,” and (7) “My athletic department takes pride in my accomplishments at work.” All seven items were scored from 0 = “strongly disagree” to 6 = “strongly agree” and averaged to form a scale of perceived organizational support (alpha = 0.93).

### Covariates

We adjust for several covariates. With respect to demographics, we controlled for coaches' age (years), gender (1 = female, 0 = male), race (white, other), education (bachelor's degree or higher = 1, all else = 0), marital status (1 = married, 0 = all else), personal income ($), and the number of children the respondent had. Several characteristics of respondents’ coaching jobs were also controlled, including whether they coached full-time (1 = yes, 0 = no), whether they coached at the NCAA Division 1 level (1 = yes, 0 = no), whether they were a head coach (1 = yes, 0 = no), the number of hours they worked per week coaching during the competitive season, and the number of career coaching jobs they had. To measure their success, coaches were also asked if they had ever won a conference championship, and if their teams had ever won a national championship.

## Statistical analyses

For each well-being outcome, a series of three models is tested. In Model 1, the association between coaching stressors and well-being is examined, net of demographic and job characteristics. Model 2 assessed the association between workplace stress and well-being, again net of all controls. Finally, Model 3 empirically tested the association between perceived organizational support and each indicator of well-being, net of all controls. Since each of the four outcome variables were continuous, we use ordinary least squares regression (OLS) in all analyses with the *p*-value set at *α* = 0.05.

## Results

Table 1 shows descriptive statistics for all study variables.

**Table 1:** Descriptive statistics, NCAA golf coaches (*N* = 48).

	Mean/%	SD	Minimum	Maximum
Dependent Variables				
Depressive Symptoms	2.14	0.37	1.63	2.88
Anxiety Symptoms	1.90	0.44	1	2.86
Burnout	2.32	1.57	0.25	5.88
Job Turnover Intentions	2.68	1.17	1	4.75
Focal Independent Variables				
Coaching Stressors Scale	3.92	1.10	1.45	6.09
Workplace Stress Scale	2.59	0.88	1.17	4.67
Perceived Organizational Support	3.53	1.66	0.25	6
Control Variables				
Age	45.81	9.27	23	64
Male	75.00			
Race (white)	91.30			
Higher than a Bachelor's Degree	41.86			
Married	78.26			
Personal Income	$79,884	$50,792.96	$35,000	$350,000
Number of Children	2.63	1.14	1	4
Full-Time Coach	84.78			
Division 1 Coach	65.22			
Head Coach	80.43			
Work Hours	56.30	14.62	20	80
Number of Career Coaching Jobs	2.79	0.99	1	4
Won a Conference Championship	50.00			
Won a National Championship	13.95			

Standard deviations are omitted for categorical variables.

Results for depressive symptoms can be found in Table 2. The coaching stressors scale did not reveal a statistically significant association with depressive symptoms (*b* = 0.04, *p *> .05) in Model 1. Model 2, however, shows that greater workplace stress was associated with higher depressive symptoms (*b* = 0.23, *p* < .05). Put in terms of effect size, this means that for every 1 unit increase in workplace stress, depressive symptoms increase by nearly 66% of a standard deviation. Model 3 also shows that greater perceived organizational support is associated with fewer depressive symptoms, net of all study covariates (*b *= −0.11, *p* < .05). Indeed, a one unit increase in perceived organizational support was associated with nearly a 33% standard deviation decrease in depressive symptoms.

**Table 2:** Results for depressive symptoms, NCAA golf coaches (*N* = 48).

	Model 1	Model 2	Model 3
Focal Independent Variables			
Coaching Stressors Scale	0.04 (0.08)		
Workplace Stress Scale		0.23 (0.08)*	
Perceived Organizational Support			−0.11 (0.05)*
Control Variables			
Age	−0.01 (0.01)	−0.01 (0.01)	−0.01 (0.01)
Male	0.04 (0.25)	0.14 (0.21)	0.11 (0.22)
Race (white)	0.27 (0.48)	0.05 (0.41)	−0.05 (0.45)
Higher than a Bachelor's Degree	0.04 (0.21)	0.07 (0.17)	0.16 (0.18)
Married	−0.36 (0.26)	−0.29 (0.21)	−0.20 (0.23)
Personal Income	0.00 (0.01)	0.00 (0.01)	0.00 (0.01)
Number of Children	0.01 (0.09)	0.02 (0.07)	0.03 (0.08)
Full-Time Coach	−0.12 (0.27)	−0.09 (0.22)	−0.06 (0.24)
Division 1 Coach	−0.07 (0.20)	−0.04 (0.16)	−0.10 (0.16)
Head Coach	0.39 (0.19)*	0.22 (0.19)	0.27 (0.20)
Work Hours	0.01 (0.01)	−0.01 (0.01)	0.01 (0.01)
Number of Career Coaching Jobs	0.15 (0.11)	0.14 (0.09)	0.16 (0.10)
Won a Conference Championship	−0.19 (0.17)	−0.14 (0.15)	−0.13 (0.16)
Won a National Championship	−0.07 (0.22)	−0.15 (0.18)	−0.10 (0.19)

Standard errors in parentheses. Unstandardized regression coefficients shown.

* *p* < .05.

Results for anxiety symptoms were similar to those for depressive symptoms. No association was found for coaching stressors, but greater workplace stress was associated with increased anxiety (*b *= 0.28, *p* < .01), corresponding to an effect size of roughly 66% of a standard deviation in anxiety symptoms. In addition, greater organizational support was associated with lower anxiety symptoms (*b* = −0.13, *p* < .01), a roughly one-third standard deviation decrease in anxiety symptoms.

Table 4 shows results for burnout. Unlike depressive and anxiety symptoms, coaching stressors were associated with higher burnout (*b *= 0.61, *p* < .05), nearly 40% of a standard deviation effect size in burnout scores, as was greater workplace stress (*b* = 1.54, *p* < .001), which corresponded to almost a full standard deviation increase in burnout scores. Similar to depressive and anxiety symptoms, higher perceived organizational support was associated with lower burnout scores (*b* = −0.59, *p* < .01), over 33% of a standard deviation decrease in burnout scores.

**Table 3:** Results for anxiety symptoms, NCAA golf coaches (*N* = 48).

	Model 1	Model 2	Model 3
Focal Independent Variables			
Coaching Stressors Scale	−0.03 (0.09)		
Workplace Stress Scale		0.28 (0.08)**	
Perceived Organizational Support			−0.13 (0.05)*
Control Variables			
Age	−0.01 (0.01)	0.01 (0.01)	−0.01 (0.01)
Male	−0.66 (0.28)*	−0.49 (0.22)*	−0.52 (0.23)*
Race (white)	0.10 (0.54)	−0.21 (0.44)	−0.35 (0.48)
Higher than a Bachelor's Degree	−0.28 (0.24)	−0.30 (0.18)	−0.19 (0.19)
Married	−0.32 (0.29)	−0.31 (0.22)	−0.20 (0.24)
Personal Income	0.00 (0.01)	0.00 (0.01)	0.00 (0.01)
Number of Children	0.21 (0.10)*	0.21 (0.08)*	0.23 (0.08)*
Full-Time Coach	0.48 (0.30)	0.50 (0.24)*	0.54 (0.25)*
Division 1 Coach	−0.29 (0.23)	−0.17 (0.16)	−0.26 (0.17)
Head Coach	0.23 (0.25)	0.05 (0.20)	0.10 (0.21)
Work Hours	0.02 (0.01)*	0.01 (0.01)	0.01 (0.01)
Number of Career Coaching Jobs	−0.15 (0.13)	−0.15 (0.10)	−0.13 (0.11)
Won a Conference Championship	−0.03 (0.19)	0.04 (0.16)	0.07 (0.17)
Won a National Championship	0.01 (0.24)	−0.09 (0.19)	−0.03 (0.20)

Standard errors in parentheses. Unstandardized regression coefficients shown.

* *p* < .05.

** *p* < .01.

**Table 4:** Results for burnout, NCAA golf coaches (*N* = 48).

	Model 1	Model 2	Model 3
Focal Independent Variables			
Coaching Stressors Scale	0.61 (0.30)*		
Workplace Stress Scale		1.54 (0.21)***	
Perceived Organizational Support			−0.59 (0.16)**
Control Variables			
Age	−0.01 (0.04)	0.01 (0.02)	−0.04 (0.03)
Male	−1.28 (0.94)	−0.46 (0.53)	−0.70 (0.80)
Race (white)	1.38 (1.96)	0.09 (1.12)	−0.21 (1.73)
Higher than a Bachelor's Degree	−1.01 (0.86)	−0.39 (0.44)	0.11 (0.68)
Married	−1.81 (1.00)	−0.79 (0.56)	−0.32 (0.87)
Personal Income	0.00 (0.01)	0.00 (0.01)	0.00 (0.01)
Number of Children	0.18 (0.33)	0.20 (0.19)	0.25 (0.28)
Full-Time Coach	−0.65 (1.08)	−0.14 (0.60)	0.01 (0.90)
Division 1 Coach	0.14 (0.75)	−0.15 (0.39)	−0.60 (0.58)
Head Coach	1.52 (0.90)	0.28 (0.52)	0.74 (0.77)
Work Hours	0.03 (0.02)	−0.01 (0.01)	0.01 (0.01)
Number of Career Coaching Jobs	0.13 (0.45)	−0.01 (0.26)	0.10 (0.38)
Won a Conference Championship	−0.60 (0.68)	−0.28 (0.38)	−0.23 (0.57)
Won a National Championship	0.34 (0.88)	−0.29 (0.47)	0.06 (0.70)

Standard errors in parentheses. Unstandardized regression coefficients shown.

* *p* < .05.

** *p* < .01.

*** *p* < .001.

### Results for job turnover intentions

Table 5 shows results for our last well-being indicator, job-turnover intentions. As with depression and anxiety, coaching stressors were not associated with greater intent to leave one's job. However, greater workplace stress (*b* = 1.05, *p* < .001) was associated with greater turnover intent, which comes close to effect size of a full one-standard deviation increase in intent for every unit increase on the workplace stress scale. Finally, greater perceived organizational support was found to be associated with lower job turnover intentions (*b* = −0.52, *p* < .001), representing just under 50% of a standard deviation in turnover intention scores.

**Table 5:** Results for job turnover intentions, NCAA golf coaches (*N* = 48).

	Model 1	Model 2	Model 3
Focal Independent Variables			
Coaching Stressors Scale	0.28 (0.24)		
Workplace Stress Scale		1.05 (0.19)***	
Perceived Organizational Support			−0.52 (0.11)***
Control Variables			
Age	−0.01 (0.03)	0.01 (0.02)	−0.03 (0.02)
Male	−0.41 (0.72)	0.33 (0.48)	0.34 (0.52)
Race (white)	1.32 (1.50)	0.28 (1.02)	−0.30 (1.13)
Higher than a Bachelor's Degree	0.03 (0.65)	0.51 (0.40)	0.95 (0.44)*
Married	−0.19 (0.80)	0.41 (0.51)	0.90 (0.57)
Personal Income	0.00 (0.01)	0.00 (0.01)	0.00 (0.01)
Number of Children	−0.17 (0.25)	−0.15 (0.17)	−0.11 (0.18)
Full-Time Coach	0.09 (0.82)	0.51 (0.54)	0.68 (0.59)
Division 1 Coach	0.03 (0.57)	−0.19 (0.35)	−0.54 (0.38)
Head Coach	0.11 (0.68)	−0.66 (0.47)	−0.48 (0.50)
Work Hours	0.02 (0.02)	−0.01 (0.01)	0.01 (0.01)
Number of Career Coaching Jobs	0.07 (0.35)	−0.01 (0.23)	0.06 (0.25)
Won a Conference Championship	0.04 (0.53)	0.35 (0.34)	0.43 (0.37)
Won a National Championship	−0.11 (0.66)	−0.65 (0.43)	−0.41 (0.46)

Standard errors in parentheses. Unstandardized regression coefficients shown.

* *p* < .05.

*** *p* < .001

## Discussion

This study explored the effects of workplace stressors and supports on the mental health of golf coaches. Our results revealed several key findings. First, coaching stressors (administrative tasks, practice plans, pressure to win, dealing with parents, time demands) were only associated with greater burnout. Initially, this was surprising because it was expected that these forms of stress would also associate with depressive and anxiety symptoms as well as job turnover based on previous literature (1, 10, 11). There are two possible interpretations of this finding. As McNeill and colleagues (16, 40) acknowledge, studies exploring depression and anxiety among coaches are rare. Thus, while previous studies may have anticipated a relationship between coaching stressors and these outcomes, there is not a large empirical basis for doing so. Moreover, it could be that some golf coaches normalize their stress level, and may under-report depressive and anxiety symptoms, which could have explained the lack of an observed association. However, our results also showed that coaching stressors aligned with burnout which was consistent with our expectations and with the existing literature. It may be that burnout is a factor that ultimately leads to depression in coaches (41). Therefore, if these coaches were studied over a longer period, one might expect coaching stressors to be associated with greater depressive and anxiety symptoms *as a result of* (i.e., mediated by) burnout, but it is currently unknown how this would unfold over time. Taken together, the divergent patterns of results suggest the importance of studying various outcomes related to well-being, as they may hold differing associations with characteristics of the job.

There was also consistent evidence that workplace stress (e.g., lack of control and autonomy, little work-family balance, little recognize, overwhelming work demands) was consistently associated with all outcomes, including greater depressive and anxiety symptoms, greater burnout, and higher job turnover intentions. Coaches who felt that they had at least some control over their work processes tended to feel better mentally and are less prone to burning out. This may be especially important to consider among golf coaches: since they are not coaching a revenue-generating sport in their athletic departments, perceive that they have some control over their work process and are fairly recognized by those around them may be even more crucial.

The last key finding of this study was, much like workplace stress, greater perceived organizational support had a consistent association with all four outcomes, but this time in a beneficial direction. Coaches who perceived that their extra efforts were noticed, felt valued and cared for by their athletic department, and found that their athletic department took part in their accomplishment reported fewer depressive and anxiety symptoms, lower scores on burnout, and lower job turnover intentions. This is an innovative finding, since most studies of coach well-being do not consider organizational factors (cf. 42–44). Though this finding would likely apply to coaches of all collegiate sports, we again highlight the somewhat lower relative status of golf coaches in athletic departments, given that they are not in a revenue-generating sport and that their competitions are rarely televised or attended by fans of the school's athletic program. This result concurs with previous research which has found that pride and organizational support are linked to a higher sense of belonging and well-being and greater job commitment (34). The consistency of salubrious associations that flow from perceived organizational support suggest that athletic departments are valuable resources that can help to promote better well-being of coaches. Though college coaches are usually held responsible for their own well-being as well as that of their student-athletes (6, 18), our study has clearly shown that organizational support can influence a range of outcomes. This suggests that any interventions to improve coach well-being (e.g., coach support groups, stress-reduction workshops; see 45) should be designed with the organization in mind.

Several study limitations must be acknowledged. First, like other studies of coach well-being, our study used a quantitative, cross-sectional design. While the current study is useful given its focus on golf coaches and extends our understanding of coach well-being and its organizational antecedents, collecting longitudinal data on coaches over an entire season to understand when mental health problems and burnout are at their highest (e.g., mid-season, end of season) and to observe how many coaches switched jobs or left the profession entirely, would also be instructive. We also lacked knowledge of other jobs that may have been held by the part-time coaches (15%), which could undoubtedly factor into assessments of mental health and burnout. It would also be helpful to assess if coaches were able to recover from burnout, how long it takes to recover, and the factors that help this process. The sample size is small, which also reduces statistical power and does not allow for the generalizability of our results. Yet, that we still observed significant associations likely suggests that are findings are conservative, and additional research with more participants might reveal the results of workplace stress to be more devastating for golf coaches than documented here. We would also hypothesize that similar results to ours would be observed among collegiate coaches in other non-revenue generating sports. Finally, future research should also control for number of years of coaching experience, another factor which might influence the patterns of results we observed.

Second, while the sample did include collegiate golf coaches at different NCAA levels and tried to achieve a reasonable gender distribution, racial diversity in golf coaches was virtually non-existent. Our sample was also not encompassing of all collegiate golf coaches, so future studies should attempt to collect larger samples of coaches to expand on our findings. Finally, future research could encompass the perspectives from the student-athlete, such as examining the experiences of athletes that play for coaches with various levels of mental well-being, or who perceive more or less organizational support. There is evidence that athletes playing for burnt out coaches reported lower athletic enjoyment, greater anxiety, and were more prone to burn out themselves (e.g., 46).

In closing, this study offers some implications of these normative levels of “fatigue” and stress symptoms that are prevalent in golf coaching. Golf coaches often have a long history of both playing and coaching in the sport, where high levels of stress and pressure are normalized. To make matters worse, elite coaches often feel vulnerable in seeking help for mental health issues or admitting struggles (47). Despite reporting at least some symptoms of depression, anxiety, and burnout, the data further show that only two of the 48 coaches studied (4.16%) had ever sought professional help (e.g., counseling, therapy). In addition to providing organizational support to coaches, it is vital that coaches have similar access to mental health resources on campus as student-athletes. Sport administrators can also help coaches to prioritize self-care strategies and set boundaries on their work (e.g., e-mail response expectations, weekly work hour commitments), as over one-quarter of our sample reported working 60 h a week or more. As the results show, this lifestyle is not without cost and is not sustainable for many coaches, despite their intense passion and commitment to their athletic programs.

## Data Availability

The raw data supporting the conclusions of this article will be made available by the authors, without undue reservation.

## References

[1] Frey M. College coaches’ experiences with stress—” problem solvers” have problems, too. Sport Psychol. (2007) 21(1):38–57. 10.1123/tsp.21.1.38

[2] Didymus FF. Olympic and international level sports coaches’ experiences of stressors, appraisals, and coping. Qual Res Sport Exerc Health. (2017) 9(2):214–32. 10.1080/2159676X.2016.1261364

[3] Dixon MA Bruening JE. Perspectives on work-family conflict in sport: an integrated approach. Sport Manage Rev. (2005) 8(3):227–53. 10.1016/S1441-3523(05)70040-1

[4] Bentzen M Lemyre PN Kenttä G. Development of exhaustion for high-performance coaches in association with workload and motivation: a person-centered approach. Psychol Sport Exerc. (2016) 22:10–9. 10.1016/j.psychsport.2015.06.004

[5] Turner BA Chelladurai P. Organizational and occupational commitment, intention to leave, and perceived performance of intercollegiate coaches. J Sport Manag. (2005) 19(2):193–211. 10.1123/jsm.19.2.193

[6] Olusoga P Maynard I Hays K Butt J. Coaching under pressure: a study of olympic coaches. J Sports Sci. (2012) 30(3):229–39. 10.1080/02640414.2011.639384 22168369

[7] Carson F Malakellis M Walsh J Main LC Kremer P. Examining the mental well-being of Australian sport coaches. Int J Environ Res Public Health. (2019) 16(23):4601. 10.3390/ijerph16234601 31756968 PMC6926512

[8] Annika Academy. How much do D1 college coaches make? Accessed at: Available at: https://www.theannikaacademy.com/how-much-do-d1-college-golf-coaches-make/ (2022).

[9] Bradford SH Keshock CM. Factors of burnout in high school coaches. J Contemp Athl. (2011) 5(1):43–53.

[10] Fletcher D Scott M. Psychological stress in sports coaches: a review of concepts, research, and practice. J Sports Sci. (2010) 28(2):127–37. 10.1080/02640410903406208 20035490

[11] Levy A Nicholls A Marchant D Polman R. Organisational stressors, coping, and coping effectiveness: a longitudinal study with an elite coach. Int J Sports Sci Coach. (2009) 4(1):31–45. 10.1260/1747-9541.4.1.31

[12] Altfeld S Schaffran P Kleinert J Kellmann M. Minimising the risk of coach burnout: from research to practice. Int Sport Coach J. (2018) 5(1):71–8. 10.1123/iscj.2017-0033

[13] Bentzen M Kenttä G Lemyre P-N. Elite football coaches’ experiences and sensemaking about being fired: an interpretative phenomenological analysis. Int J Environ Res Public Health. (2020) 17(14):5196. 10.3390/ijerph17145196 32708462 PMC7400468

[14] Pawsey F Wong JHK Kenttä G Näswall K. Daily mindfulness is associated with recovery processes among coaches—a 4-week diary study. Int Sport Coach J. (2021) 8(3):371–81. 10.1123/iscj.2020-0045

[15] Potrac P Smith A Nelson L. Emotions in sport coaching: an introductory essay. Sports Coach Rev. (2017) 6(2):129–41. 10.1080/21640629.2017.1375187

[16] McNeill K Durand-Bush N Lemyre PN. Understanding coach burnout and underlying emotions: a narrative approach. Sports Coach Rev. (2017) 6(2):179–96. 10.1080/21640629.2016.1163008

[17] Kelley BC Eklund RC Ritter-Taylor M. Stress and burnout among collegiate tennis coaches. J Sport Exerc Psychol. (1999) 21(2):113–30. 10.1123/jsep.21.2.113

[18] Thelwell RC Weston NJ Greenlees IA Hutchings NV. Stressors in elite sport: a coach perspective. J Sports Sci. (2008) 26(9):905–18. 10.1080/02640410801885933 18569556

[19] Olusoga P Butt J Hays K Maynard I. Stress in elite sports coaching: identifying stressors. J Appl Sport Psychol. (2009) 21(4):442–59. 10.1080/10413200903222921

[20] Durand-Bush N Collins J McNeill K. Women coaches’ experiences of stress and self-regulation: a multiple case study. Int J Coach Sci. (2012) 6(2):21–43.

[21] Mignano MJ. Organizational factors and coach burn-out in collegiate athletics. East Lansing, MI: Michigan State University (2020).

[22] Potts AJ Didymus FF Kaiseler M. Psychological stress and psychological well-being among sports coaches: a meta-synthesis of the qualitative research evidence. Int Rev Sport Exerc Psychol. (2021), 10.1080/1750984X.2021.1907853.

[23] Corsby CL Jones R Lane A. Contending with vulnerability and uncertainty: what coaches say about coaching. Sports Coach Rev. (2023) 12(3):323–42. 10.1080/21640629.2022.2057697

[24] Orr S Carson HJ Cruickshank A. How do coaches operationalise long-term technical training in elite golf? Int Sport Coach J. (2022) 9(3):319–30. 10.1123/iscj.2021-0059

[25] Maslach C Leiter MP. The truth about burnout: How organizations cause personal stress and what to do about it. San Francisco, CA: Jossey-Bass (1997).

[26] Freudenberger HJ. Staff burn-out. J Soc Issues. (1974) 30(1):159–65. 10.1111/j.1540-4560.1974.tb00706.x

[27] Pervez A Halbesleben J. Burnout and well-being. In: Burke RJ Page KM, editors. Research handbook on work and well-being. Cheltenham, United Kingdom: Edward Elgar (2017). p. 101–22.

[28] Raedeke TD. Coach commitment and burnout: a one-year follow-up. J Appl Sport Psychol. (2004) 16(4):333–49. 10.1080/10413200490517995

[29] Raedeke TD Lunney K Venables K. Understanding athletes’ burnout: coach perspectives. J Sport Behav. (2002) 25(2):181–206.

[30] Raedeke TD Granzyk TL Warren A. Why coaches experience burnout: a commitment perspective. J Sport Exerc Psychol. (2000) 22(1):85–105. 10.1123/jsep.22.1.85

[31] Mclaine AJ. An overview of burnout in athletic trainers. Int J Athl Ther Train. (2005) 10(6):11–3. 10.1123/att.10.6.11

[32] Ryff CD Keyes CLM. The structure of psychological well-being revisited. J Pers Soc Psychol. (1995) 69(4):719–27. 10.1037/0022-3514.69.4.719 7473027

[33] Ha JP Ha J. Organizational justice–affective commitment relationship in a team sport setting: the moderating effect of group cohesion. J Manag Organ. (2015) 21(1):107–24. 10.1017/jmo.2014.67

[34] Aube C Rousseau V Morin EM. Perceived organizational support and organizational commitment: the moderating effect of locus of control and work autonomy. J Manag Psychol. (2007) 22(5):479–95. 10.1108/02683940710757209

[35] Cunningham GB Sagas M. The differential effects of human capital for male and female division I basketball coaches. Res Q Exerc Sport. (2002) 73(4):489–95. 10.1080/02701367.2002.10609051 12495253

[36] Burnam MA Wells KB Leake B Landsverk J. Development of a brief screening instrument for detecting depressive disorders. Med Care. (1988) 26(8):775–89. 10.1097/00005650-198808000-00004 3398606

[37] Pallant JF Tennant A. An introduction to the rasch measurement model: an example using the hospital anxiety and depression scale (HADS). Br J Clin Psychol. (2007) 46(1):1–18. 10.1348/014466506X96931 17472198

[38] Kokkinos CM. Factor structure and psychometric properties of the maslach burnout inventory-educators survey among elementary and secondary school teachers in cyprus. Stress Health. (2006) 22(1):25–33. 10.1002/smi.1079 28066969

[39] Roodt G. Concept redundancy and contamination in employee commitment research: current problems and future directions. SA J Ind Psychol. (2004) 30(1):82–90. 10.4102/sajip.v30i1.135

[40] McNeill K Durand-Bush N Lemyre PN. Thriving, depleted, and at-risk Canadian coaches: profiles of psychological functioning linked to self-regulation and stress. Int Sport Coach J. (2018) 5(2):145–55. 10.1123/iscj.2017-0042

[41] Bianchi R Schonfeld IS Laurent E. Burnout–depression overlap: a review. Clin Psychol Rev. (2015) 36:28–41. 10.1016/j.cpr.2015.01.004 25638755

[42] Dixon MA Sagas M. The relationship between organizational support, work-family conflict, and the job-life satisfaction of university coaches. Res Q Exerc Sport. (2007) 78(3):236–47. 10.1080/02701367.2007.10599421 17679497

[43] Kim J Cunningham GB. Moderating effects of organizational support on the relationship between work experiences and job satisfaction among university coaches. Int J Sport Psychol. (2005) 36(1):50–64.

[44] Rocha CM Chelladurai P. Relationship between organizational support and performance of college coaches: a mediational model. Eur Sport Manag Q. (2011) 11(3):301–19. 10.1080/16184742.2011.577793

[45] Dreison KC Luther L Bonfils KA Sliter MT McGrew JH Salyers MP. Job burnout in mental health providers: a meta-analysis of 35 years of intervention research. J Occup Health Psychol. (2018) 23(1):18–30. 10.1037/ocp0000047 27643608

[46] Price MS Weiss MR. Relationships among coach burnout, coach behaviors, and athletes’ psychological responses. Sport Psychol. (2000) 14(4):391–409. 10.1123/tsp.14.4.391

[47] Olusoga P Kenttä G. Desperate to quit: a narrative analysis of burnout and recovery in high-performance sports coaching. Sport Psychol. (2017) 31(3):237–48. 10.1123/tsp.2016-0010

